# The Protective Effect of *Panax notoginseng* Mixture on Hepatic Ischemia/Reperfusion Injury in Mice *via* Regulating NR3C2, SRC, and GAPDH

**DOI:** 10.3389/fphar.2021.756259

**Published:** 2021-11-11

**Authors:** Wen Hou, Bao Wei, Hong Sheng Liu

**Affiliations:** ^1^ NHC Key Laboratory of Critical Care Medicine, Tianjin First Central Hospital, Tianjin, China; ^2^ Department of Surgery, Children’s Hospital, Tianjin, China

**Keywords:** mineralocorticoid receptor, glyceraldehyde-3-phosphate dehydrogenase liver, tyrosine-protein kinase, notoginseng mixture, hepatic ischemia/reperfusion injury

## Abstract

*Panax notoginseng* mixture (PNM) has the characteristics of multicomponent, multitarget, and multieffect, which can cope with the multidirectional and multidimensional complex pathological process caused by hepatic ischemia/reperfusion injury (HIRI). Our animal experiments showed that PNM composed of notoginseng, dogwood, and white peony root could significantly reduce the level of aspartate transaminase and alanine aminotransferase in the blood of mice with HIRI, indicating that this preparation had a protective effect on HIRI in mice. Therefore, on this basis, the molecular mechanism of PNM intervention in HIRI was further explored by network pharmacology. First, target genes corresponding to active components and HIRI were obtained through databases such as TCMSP, Pharm Mapper, Swiss Target Prediction, GeneCards, and so on. All target genes were standardized by Uniprot database, and a total of 291 target genes with their intersection were obtained. Then, Kyoto Encyclopedia of Genes and Genomes (KEGG) pathways and biological processes (BPs) of 291 target genes were obtained through the online public platform of DAVID. A total of 177 KEGG pathways and 337 BPs were obtained by setting *p* < 0.01 and false discovery rate <0.05. The network mapping map of components and disease targets was drawn by Cytoscape, and the top 10 Hub target genes related to HIRI were obtained. At the same time, the String database was used to obtain the protein–protein interaction dataset, which was imported into Cytoscape, and the first 10 Hub target genes were obtained. The Hub target genes obtained by the above two methods were molecular docking with their corresponding small molecule compounds through DockThor online tool. The results showed that the docking of paeoniflorin with glyceraldehyde 3-phosphate dehydrogenase (GAPDH), paeoniflorin and loganin with SRC, ginsenoside Rb1 with NR3C2, ursolic acid and oleanolic acid with IL-6, paeoniflorin docking VEGFA, and MMP9. Finally, NR3C2, SRC, and GAPDH were identified as target genes in this study by referring to relevant literature reports. After verification by immunohistochemical experiments, compared with the sham group, the above three target genes were highly expressed in the HIRI group (*p* < 0.01). Compared with the HIRI group, the expression of three target genes in the PNM + HIRI group was significantly decreased (*p* < 0.01). The results showed that PNM could protect mouse HIRI by decreasing the expression of NR3C2, SRC, and GAPDH.

## Introduction

Hepatic ischemia/reperfusion injury (HIRI) refers to the process of hepatic cells undergoing different degrees of apoptosis and necrosis during blood reperfusion after the temporary loss of blood supply, which aggravates hepatic function damage. It is commonly seen in the process of hepatic transplantation, hepatic resection, hemorrhagic shock or trauma, and so on (Ito et al., 2019; Zhang et al., 2019). HIRI is a key factor in hepatic failure and death after surgery (Kim and Ha, 2010). Therefore, HIRI has been one of the hot topics that medical researchers have been exploring and solving in recent years. It is very important to find ways to reduce the adverse consequence of HIRI for improving the success rate of hepatic surgery.

HIRI is a common pathophysiological phenomenon in clinical practice, and its pathogenesis remains unknown. There is no good prevention and treatment method (Shinoda et al., 2002). In recent years, intervention methods for HIRI mainly include ischemic preconditioning, drug intervention, monoclonal antibodies, signaling pathway inhibitors, cytokine antagonists, gene knockout and RNA interference, and so on (Peng et al., 2015). At present, the mechanisms of HIRI mainly include oxidative stress, complement activation, inflammatory cell infiltration, release of inflammatory factors, and cell apoptosis (Lee et al., 2014; Liu et al., 2015; Kim et al., 2016). However, most of these research ideas and methods are still in the stage of research and development, and there is still a long way to go before they can be really used in clinical practice to weaken or eliminate the efficacy of HIRI.

Recent studies have shown that monomer components of traditional Chinese medicine as pretreatment drugs play a certain mitigating role in the development of HIRI. Lin reported that ginsenoside Rg1 improved HIRI by inhibiting the mitochondrial apoptosis pathway mediated by cyclophilin D protein (Lin et al., 2020). Jawan et al. reported that magnolol had a protective effect on warm HIRI by upregulation of antiapoptotic Bcl-XL gene and inhibition of Bcl-XS gene (Jawan et al., 2003). Li et al. reported that galangin alleviated HIRI in rats by mediating the PI3K/Akt pathway (Li et al., 2018), Jiang et al. reported that oxymatrine played a protective role on warm HIRI by inhibiting apoptosis (Jiang et al., 2005). Zhang et al. reported that *Atractylodes macrocephala* on polysaccharides inhibited the expression of nuclear factor κB, inhibition of enzyme activity, and reduction of the generation of oxygen free radicals reducing HIRI (Zhang et al., 2010). However, HIRI is a complex pathological process, and its pathogenesis is multifaceted. Therefore, the study on the mechanism of a single component corresponding to a single target cannot completely solve the negative effects of HIRI in liver surgery, that is, the limited drug action model of a single component corresponding to a single target. However, the multicomponent, multitarget, and multieffect characteristics of traditional Chinese medicine exactly correspond to the multidimensional and multidimensional complex pathological process caused by HIRI; thus, this study was conducted.

The concept of network pharmacology was proposed by the British pharmacologist Hopkins (2007), which clarified that the occurrence of diseases is the result of the disruption of the dynamic balance of the interactions of multiple genes, multifunctional proteins, and multipathways in the human body; that is, the molecular basis for the occurrence of diseases is multidimensional. It is suggested that the mechanism of some single chemical drugs acting on a single disease target is not comprehensive enough. Network pharmacology can analyze the characteristics of a single component of a drug acting on different targets, cells, and organs at the molecular and genetic levels and systematically predict and reveal the action and mechanism of a drug, so as to evaluate the efficacy and adverse reactions of a drug and find new drugs with high efficiency and low toxicity. Therefore, this research method has brought great opportunities and hopes for the research and development of traditional Chinese medicine (Wang et al., 2021).

*Panax notoginseng* mixture (PNM) is based on the advice of senior clinical experts of traditional Chinese medicine and reference to the relevant Chinese medicine pharmacology books. The whole formula consists of notoginseng radix et rhizoma (notoginseng), corni fructus (dogwood), and Paeoniae radix alba (white peony root). Li et al. reported that the TCM syndrome differentiation diagnosis of HIRI was mainly blood stasis syndrome (Li, 2008). Notoginseng is a plant of the Araliaceae family, with the effect of dispersing stasis and relieving pain, reducing swelling, and relieving pain. Since ancient times, its remarkable effect of promoting blood circulation and removing blood stasis has been reputed as “priceless” (Yang et al., 2020). Thus, the effect of notoginseng on promoting blood circulation is very strong. White peony root is derived from the root of herbaceous peony cultivated in Ranunculaceae. It has the functions of nourishing blood, softening liver, and relieving pain. It is the best medicine for the treatment of chest, abdomen, waist, and rib pain in Chinese folks (Wu, 1985). Pharmacological studies have shown that white peony root has immunomodulatory, hepatoprotective, vascular dilatation, anti-inflammatory, and other effects (Wang et al., 1999). Dogwood is derived from the dried and mature pulp of *Cornus officinalis* and has the effect of tonifying the liver and kidney. Modern pharmacology shows that dogwood has functions such as liver protection, antioxidant effects, neuroprotection, and myocardial protection (Nan et al., 2018). Therefore, the efficacy of notoginseng and white peony root for promoting blood circulation and removing blood stasis, for softening the liver and relieving pain, and the efficacy of dogwood for tonifying liver and kidney and regulating immunity were combined to observe the efficacy of intervening mouse HIRI by PNM.

**FIGURE 1:**
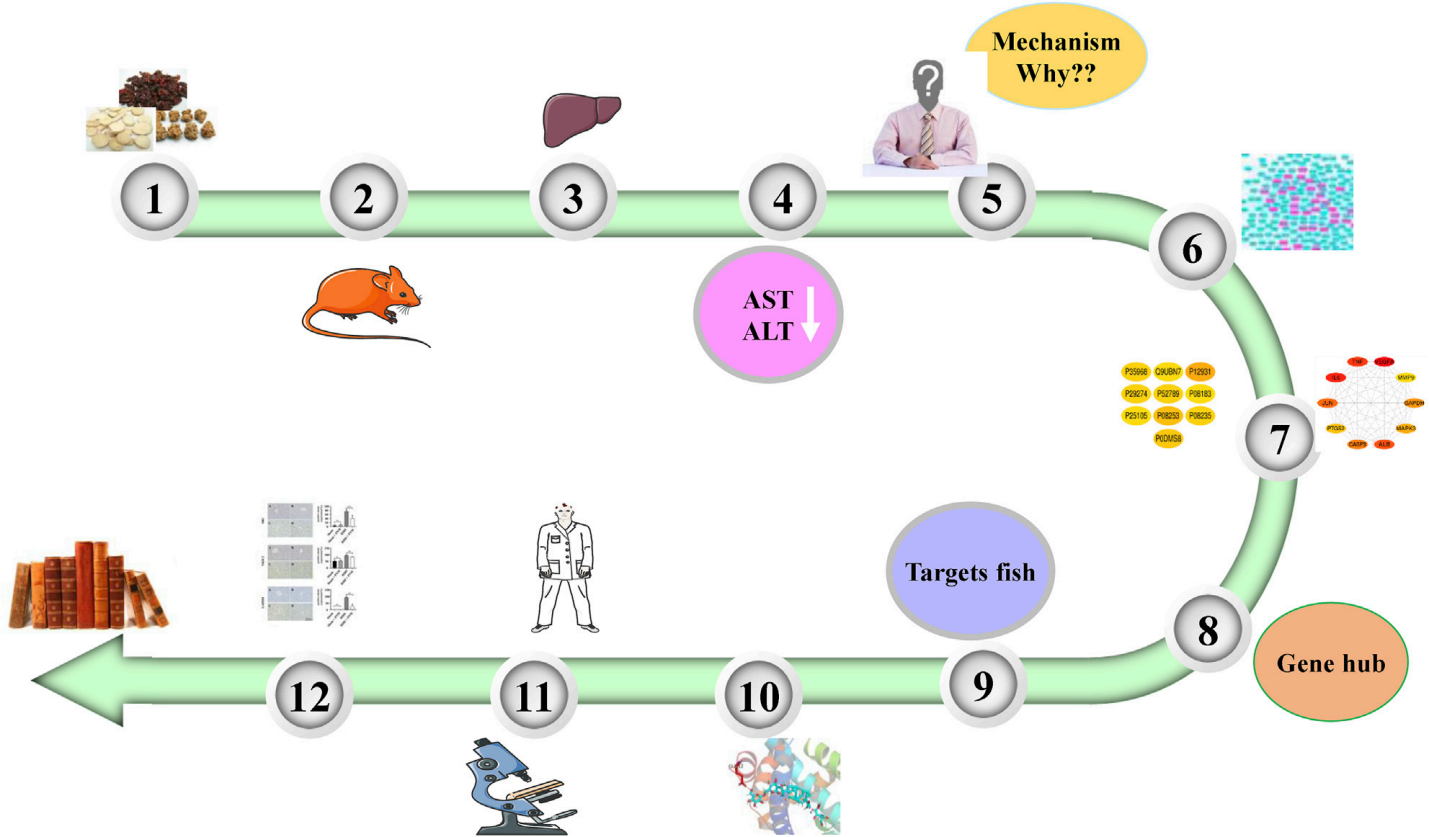
Overall design of experiment. PNM can reduce the levels of AST and ALT in HIRI mice. Therefore, the molecular mechanism was explored by using network pharmacology. First, 20 Hub target genes were screened out, and then the molecular docking technology was used to make the active site of Hub target gene receptor form a complex with its corresponding small molecule ligand by hydrogen bond. Finally, NR3C2, SRC, and GAPDH were identified as the target genes and verified by experiments.

The animal experiments showed that PNM could effectively reduce the expressions of aspartate transaminase (AST) and alanine aminotransferase (ALT) in the blood of HIRI mice model group, and there was a significant difference compared with the sham group (*p* < 0.01), indicating that PNM could effectively intervene in the occurrence of HIRI in mice. Therefore, the potential mechanism of PNM alleviating HIRI in mice using bioinformation network pharmacology is described in detail in the following. The overall design of this study is shown in Figure 1.

## Materials and Methods

### Animals and Drug Treatment

The animals used in our study were obtained from the Institute of Medical Laboratory Animals, Chinese Academy of Medical Sciences (Beijing, China), license no. SCXK (Beijing) 2014-0004. Male C57BL mice, weighing 19–21 g and aged 5–6 weeks, were raised in a specific pathogen-free environment with air-conditioning at a controlled temperature of 23.5°C ± 1.0°C and a relative humidity of 65 ± 20%. The mice were fed optionally with laboratory chow and water.

PNM (containing loganin 2.502 mg/L and paeoniflorin 6.422 mg/L) was provided by the Key Laboratory of Critical Care Emergency Medicine of the National Health Commission (Tianjin, China). It was administered intragastrically (10 mg/g, raw drug/body weight of mice).

### Animal Surgery

Twenty-eight mice were randomly divided into four groups with seven mice in each group, which were sham, sham + PNM, HIRI, and HIRI + PNM, respectively. Among them, sham + PNM and HIRI + PNM were given intragastric administration PNM for 14 days, once a day. The other sham and HIRI were given the same amount of normal saline by intragastric administration. One hour after the last administration of preconditioning, animals were anesthetized by injection intraperitoneally with pentobarbital sodium (40 ng/g). The abdominal cavity is opened in the middle of the upper abdomen, carefully exposing the liver, and the hilar ligament is dissociated. In HIRI and HIRI + PNM groups, the left lobe and middle lobe of the liver were clipped to block blood flow, and the clipping was loosened 1 h later to restore blood flow. The sham group only dissociated the hilum without blocking the blood flow. Six hours after reperfusion, eyeballs were removed for blood collection, and parts of the liver tissues were separated and stored at −80°C, whereas the remaining liver tissues were stored in formalin.

### Blood Biochemical Analyses and Liver Histological Examination

The collected blood was placed at room temperature for 60 min and centrifuged at 3,000 revolutions/min for 20 min. The serum was separated, and AST and ALT in the blood of mice were determined using an automatic clinical biochemical analyzer (Sysmex Chemix-180) from the First Central Hospital of Tianjin, China. Liver specimens for histopathological analysis were obtained 6 h after reperfusion. Samples were fixed in 4% paraformaldehyde buffer solution and then embedded in paraffin. The samples were sliced into 5-μm sections, dewaxed with xylene and ethanol, and stained with hematoxylin and eosin (HE), followed by dehydration. Then the slices were observed with microscope inspection. Histological changes were evaluated in randomly chosen histological fields at 400× magnification.

### Collection, Screening, and Target Prediction of Active Components of PNM

In traditional Chinese medicine database and analysis platform system pharmacology TCMSP (http://tcmspw.com./tcmsp.php), refer to the related literature for collecting the chemical component information of notoginseng, dogwood, and white peony root in PNM formula. Oral bioavailability (OB) ≥30% and drug-like drug (DL) ≥0.18 (Zhang M. et al., 2020) were the screening criteria for obtaining small molecule compounds, and applying the PharmMapper (http://lialb-ecust.cn/pharmmapper/) and Swiss Target Prediction (http://www.swisstarget.progress.ch/) database predicts the corresponding target genes. After the elimination of duplication, the target genes were unified in the Uniprot (http://www.uniprot.org/) service platform for correction and transformation and finally represented by Uniprot ID to establish the target database of active ingredients of PNM.

### Construction of Active Ingredient-HIRI Target Gene Network

In GeneCards (http://www.genecards.org/) database, “hepatic ischemia reperfusion injury” was used as keywords to predict the target genes associated with HIRI, and correction and transformation were performed in the Uniprot service platform. Finally, it is represented by Uniprot IDs. A total of 291 target genes with the intersection of components and HIRI were collected by using the function of finding duplicates in Excel, and their Uniprot IDs were imported into Cytoscape 3.7.2 software to construct a visual network diagram of active components and target genes of HIRI disease. Then, the first 10 Hub target genes were obtained by using the MCC function of Cytoscape 3.7.2 plug-in cytoHubba.

### Protein–Protein Interaction Analysis of Target Gene With Intersection

Gene symbol of 219 target genes with intersection was imported into the String (https://string-db.org/) service platform, and the limited species was *Homo sapiens* to construct a protein–protein interaction (PPI) network. Meanwhile, the PPI dataset was imported into Cytoscape 3.7.2, and the top 10 Hub target genes of PPI were obtained by using the MCC function of cytoHubba plug-in.

### Kyoto Encyclopedia of Genes and Genomes Pathway and Biological Processes Analysis

The Kyoto Encyclopedia of Genes and Genomes (KEGG) and biological process (BP) analysis were performed using DAVID (https://david.ncifcrf.gov/) on 291 target genes with the OFFICIAL_GENE SYMBOL as the select identifier, and the species as *H. sapiens*. KEGG and BP enrichment datasets were obtained using *p* < 0.01 and false discovery rate (FDR) <0.05 as screening conditions, and their results were presented by bar plots.

### Molecular Docking Analysis

First, the Hub target genes (Hub target genes obtained through MCC and PPI) and their corresponding active components were applied to PDB (http://www.rcsb.org/) and the PubChem database (https://pubchem.ncbi.nlm.nih.gov/) to download the structure of protein and small molecule compounds and use the DockThor (https://dockthor.lncc.br/v2/) online tool for molecular docking. According to the molecular docking results and literature review, three target genes, NR3C2, SRC, and glyceraldehyde 3-phosphate dehydrogenase (GAPDH), were finally selected for experimental verification to reveal the molecular mechanism of PNM intervention in HIRI.

### Immunohistochemistry

First, the slices were successively put into xylene, anhydrous ethanol, and distilled water to complete the dewaxing of paraffin slices to water. The tissue sections were then placed in a box filled with citric acid (pH 6.0) solution and put into a microwave oven for antigen repair. The box was heated for 8 min to boil, then held fire for 8 min, and then heated at medium and low heat for 7 min. After natural cooling, the sections were placed in PBS (pH 7.4) and washed by shaking on a decolorizing bed for three times, 5 min each. The slices were then incubated in 3% hydrogen peroxide solution at room temperature and protected from light for 25 min to block endogenous peroxidase. Then, 3% bovine serum albumin was added evenly in the section ring and sealed at room temperature for 30 min. Then, the blocking fluid was gently removed; PBS diluted antibody (NR3C2 [1:500]), SRC (1:500), and GAPDH (1:1,000) were added to the sections, respectively. The sections were placed flat in the wet box and incubated overnight at 4°C. On the second day, the sections were washed with PBS, and the secondary antibody (horseradish peroxidase label) corresponding to the primary antibody was added to cover the tissues in the circle, and the tissues were incubated at room temperature for 50 min. Then, after washing the slices with PBS, the newly prepared DAB chromogenic solution was added into the ring. The chromogenic time was controlled under the microscope, and the positive color was brown-yellow. The chromogenic process was terminated by washing the slices with tap water. Finally, the nuclei were recolored with hematoxylin and sealed by dehydration.

### Data Analysis

Immunohistochemical assay was performed to quantitatively analyze the positive staining intensity of 3 sections (*n* = 3) in each group by histochemistry score (H-score) method. One-way analysis of variance and Tukey *post hoc* statistics were used to analyze the differences between groups. *p* < 0.05 was considered statistically significant.

## Results

### Effects of PNM on AST and ALT in Serum of HIRI Mice

**FIGURE 2:**
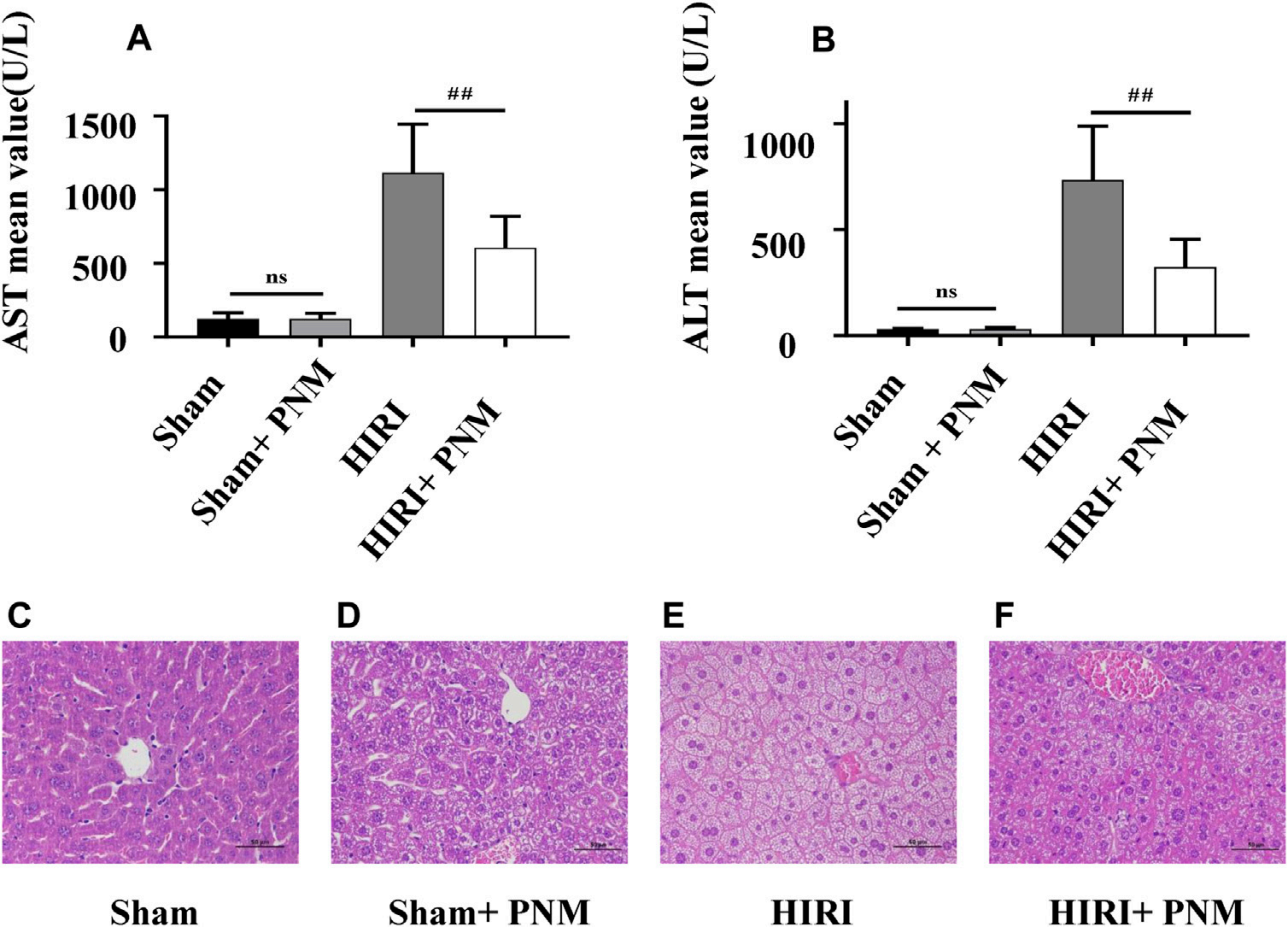
Effects of PNM on AST and ALT in serum of HIRI mice. Figure **(A,B)** shows that the expressions of AST and ALT in serum of mice in the HIRI group were significantly higher than those in the sham group (*p* ˂ 0.01), indicating successful modeling. Compared with the HIRI group, the expressions of AST and ALT in the serum of mice in the HIRI + PNM group were significantly decreased (^##^ *p* ˂ 0.01), indicating that PNM had a protective effect on HIRI in mice. There was no significant difference in serum AST and ALT between the sham and sham + PNM groups (*p* > 0.05), indicating that PNM does not cause damage to healthy mice, which proves the safety of PNM (n = 7). **(C–F)** Results of HE staining experiment. **(C)** The mouse liver tissue was intact. **(D)** Liver tissue was largely intact. **(E)** Liver tissue showed varying degrees of swelling/necrosis, steatosis, inflammatory cell infiltration, and so on. **(F)** The pathological changes of mouse liver tissue were improved, indicating that PNM had a protective effect on HIRI mice. The scale is 50 μm, and the magnification is 400 times.

Mice were randomly divided into four groups, with seven mice in each group, which were sham, sham + PNM, HIRI, and HIRI + PNM, respectively. Sham + PNM and HIRI + PNM groups were given intragastric administration PNM for 14 days, once a day, whereas the sham and HIRI groups were given normal saline by intragastric administration for 14 days, once a day. For the HIRI and HIRI + PNM groups, operation was performed for 1-h liver ischemia and 6-h reperfusion, whereas for the sham and sham + PNM group, only the hilum was dissociated without blocking the blood flow. The serum samples of the four groups of animal experiments were detected by automatic clinical biochemical analyzer; compared with the sham group, the serum ALT and AST levels in the HIRI model group were significantly increased (*p* < 0.01), indicating successful modeling. Compared with the HIRI model group, the serum ALT and AST levels in the PNM group were significantly decreased (*p* < 0.01), indicating that PNM had a protective effect on HIRI mice. There was no statistical significance in the changes of ALT and AST in the serum between the sham and sham + PNM groups, indicating that PNM would not cause damage to normal mice, proving the safety of PNM (Figures 2A,B). At the same time, HE staining was performed on the liver tissue sections of mice. The results of the four groups showed that, compared with the sham group, the liver tissue of mice in the HIRI group showed swelling/necrosis, steatosis, inflammatory cell infiltration, and so on, at different degrees. Compared with the HIRI group, the pathological changes of liver tissue of mice in the HIRI + PNM group were improved, indicating that PNM had a protective effect on HIRI in mice. The liver tissues of mice in the sham and sham + PNM groups were basically unchanged, indicating that PNM basically did not cause damage to the liver tissues of normal mice (Figures 2C–F).

### Collection, Screening, and Target Prediction of Active Components of PNM

Through TCMSP database and taking OB ≥30% and DL ≥0.18 as screening conditions, the active ingredients of the three drugs were obtained. Among them, notoginseng has 9 components, dogwood has 20 components, and white peony root has 11 components. In addition, according to literature reports, candidates of active ingredients that were not included in the screening criteria but had important biological activities and pharmacological effects were also incorporated (Chen et al., 2019) including three components of notoginseng, three components of dogwood, and four components of white peony root. After combining the common ingredients, a total of 41 active ingredients were finally obtained (Table 1).

**TABLE 1:** Basic information of PNM compounds.

Compound code	Compound name	OB/%	DL	Medicine
MOL008457	Tetrahydroalstonine	32.42	0.81	Dogwood
MOL007487	Notoginsenosider1	5.42	0.13	Notoginseng
MOL007488	Notoginsenosider2	7.69	0.28	Notoginseng
MOL007476	Ginsenoside Rb1	6.29	0.04	Notoginseng
MOL007475	Ginsenoside F2	36.43	0.25	Notoginseng
MOL005531	Telocinobufagin	69.99	0.79	Dogwood
MOL005530	Hydroxygenkwanin	36.47	0.27	Dogwood
MOL005503	Cornudentanone	39.6	0.33	Dogwood
MOL005489	3,6-Digalloylglucose	31.42	0.66	Dogwood
MOL005486	3,4-Dehydrolycopen-16-al	46.64	0.49	Dogwood
MOL005481	2,6,10,14,18-Pentaene	33.40	0.24	Dogwood
MOL005360	Malkangunin	57.71	0.63	Dogwood
MOL005344	Ginsenoside rh2	36.32	0.56	Notoginseng
MOL003137	Leucanthoside	32.12	0.78	Dogwood
MOL002883	Ethyl oleate (NF)	32.40	0.19	Dogwood
MOL002879	Diop	43.59	0.39	Dogwood, notoginseng
MOL001933	Oxypaeoniflorin	21.88	0.78	White peony root
MOL001930	Benzoyl paeoniflorin	31.77	0.75	White peony root
MOL001928	Albiflorin_qt	66.64	0.33	White peony root
MOL001927	Albiflorin	12.09	0.77	White peony root
MOL001925	Paeoniflorin_qt	68.18	0.40	White peony root
MOL001924	Paeoniflorin	53.87	0.79	White peony root
MOL001921	Lactiflorin	49.12	0.80	White peony root
MOL001911	Albiflorin R1	21.29	0.82	White peony root
MOL000874	Paeonol	28.74	0.04	White peony root
MOL001792	DFV	32.76	0.18	Notoginseng
MOL001771	Poriferast-5-en-3β-ol	36.91	0.75	Dogwood
MOL001680	Loganin	59.00	0.44	Dogwood
MOL001495	Ethyl linolenate	46.10	0.20	Dogwood
MOL001494	Mandenol	42.00	0.19	Dogwood, notoginseng
MOL000554	Gallicacid-3-O-(6′-O-galloyl)-glucoside	30.25	0.67	Dogwood
MOL000511	Ursolic acid	16.77	0.75	Dogwood
MOL000492	(+)-Catechin	54.8	0.24	White peony root
MOL000449	Stigmasterol	43.8	0.75	Dogwood, notoginseng
MOL000422	Kaempferol	41.88	0.24	White peony root
MOL000359	Sitosterol	36.91	0.75	White peony root
MOL000358	β-Sitosterol	36.90	0.75	White peony root, dogwood, notoginseng
MOL000263	Oleanolic acid	29.02	0.76	White peony root, dogwood
MOL000211	Mairin	55.38	0.24	White peony root
MOL000098	Quercetin	46.43	0.28	Notoginseng
MOL000069	Palmitic acid	19.30	0.10	Dogwood

The chemical composition structure was uploaded to the Pharm Mapper service platform in MOL2 format for target prediction. The SMILES of chemical composition is uploaded to the Swiss Target Prediction database platform for target prediction. After collecting targets, sorting out, and removing duplicate items, they were represented by Uniprot ID. Finally, a total of 2,677 predicted targets corresponding to the components of three drugs were obtained.

### Construction of Active Ingredient–HIRI Target Gene Network and Acquisition of Hub Target

**FIGURE 3:**
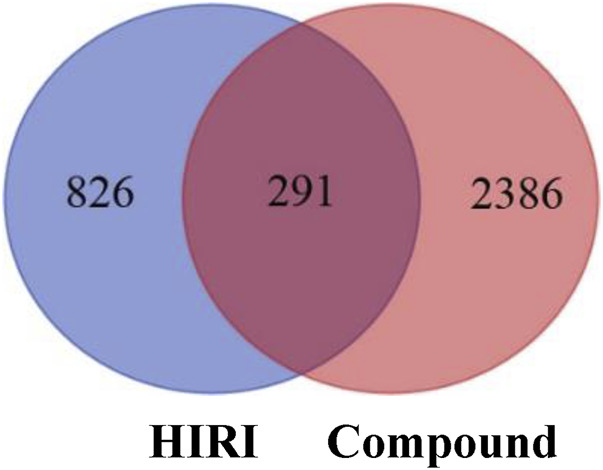
Intersection diagram of components and disease targets. The blue part in the figure represents the number of HIRI target genes obtained, that is, 1,117, and the red part represents the number of constituent target genes obtained, that is, 2,677. There were 291 target genes in the intersection between components and HIRI.

**FIGURE 4:**
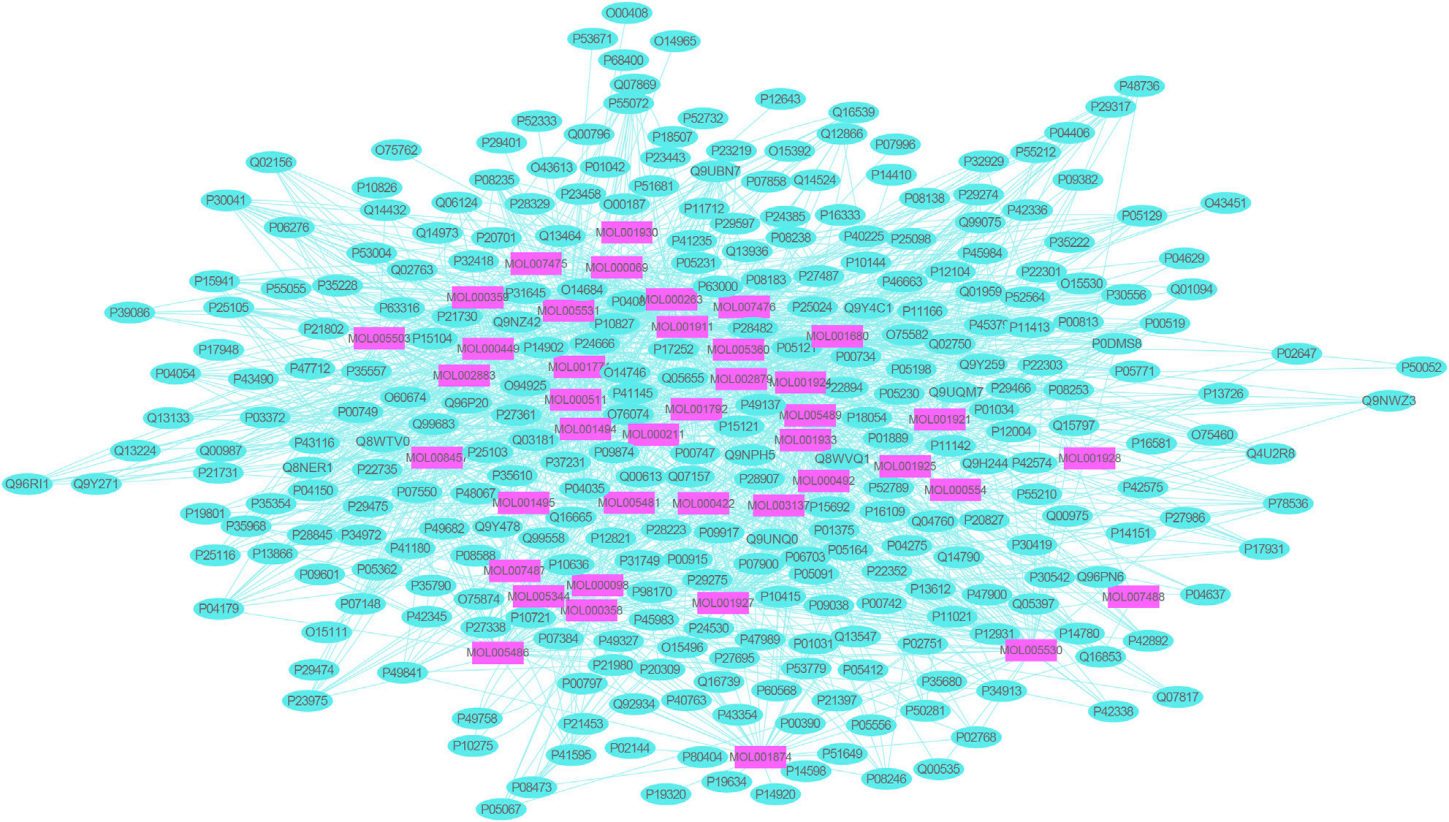
Network diagram of mutual mapping between components and HIRI. In the figure, the pink rectangle represents the component target gene, the blue oval represents the HIRI target gene, and the light blue color line represents the intersection line between the component and the target gene.

**FIGURE 5:**
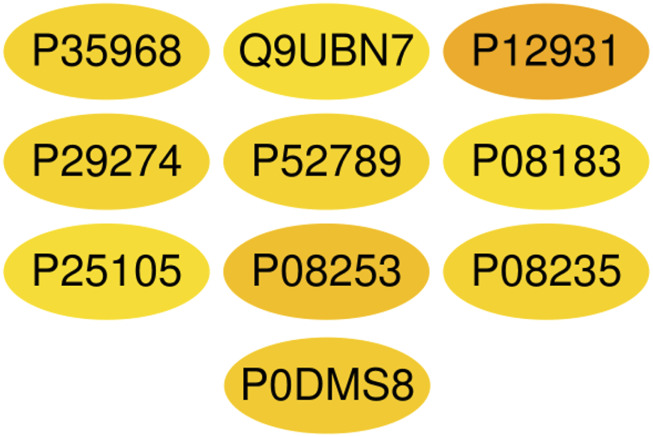
Top 10 Hub target genes of HIRI obtained through the MCC function. The color gradually changes from yellow to red; the closer to the red target gene, the more important it is. They are, respectively, P15692 (VEGFA), P05231 (IL-6), P01375 (TNF), P02768 (Alb), P05412 (Jun), P42574 (Casp3), P04406 (GAPDH), MAPK3 (P27361), P35354 (PTGS2), and MMP9 (P14780).

A total of 1,117 target genes were obtained from Genecards database based on the keywords of “hepatic ischemia reperfusion injury” and expressed as Uniprot ID.2677 components, and from the predicted target genes in the intersection, 291 intersection target genes were obtained, and the Venn diagram (http://bioinformatics.psb.ugent.be/webtools/Venn/) software was used for its visualization, as shown in (Figure 3). The composition–HIRI visual network diagram was drawn by using Cytoscape 3.7.2 software. A pink rectangle represents the composition, a blue oval represents the target gene, and a light blue line represents the intersection line between the component and the target gene (Figure 4). Then, the first 10 Hub target genes were obtained by using the MCC function of Cytoscape3.7.2 plug-in cytoHubba. They were P12931 (SRC), P08253 (MMP2), P0DMS8 (ADORA3), P35968 (KDR), P52789 (HK2), P29274 (ADORA2a), P08235 (NR3C2), P25105 (PTAFR), P08183 (ABCB1), and Q9UBN7 (HDAC6), as shown in (Figure 5).

### Hub Target Genes Were Obtained From the String Database

**FIGURE 6:**
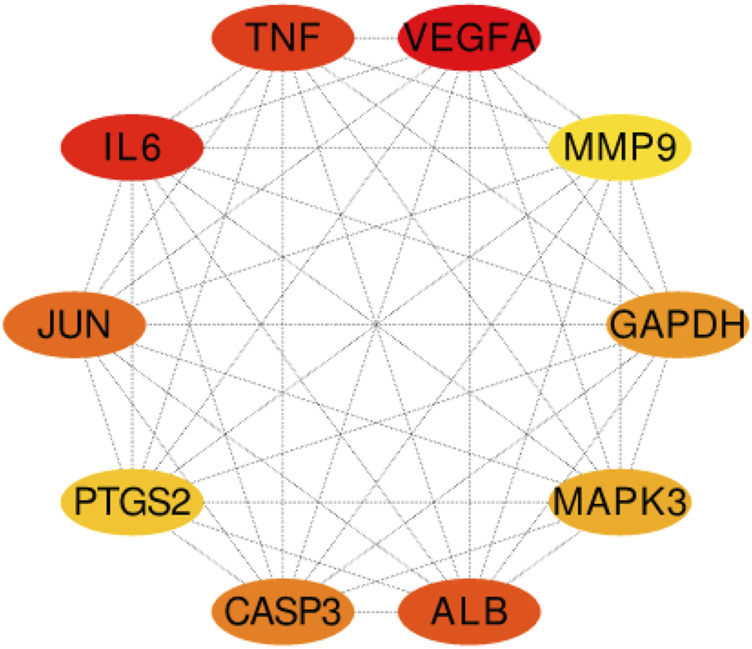
The top 10 Hub target genes of PPI were obtained through MCC function. The color gradually changes from yellow to red; the closer to the red target gene, the more important it is. They are, respectively, P15692 (VEGFA), P05231 (IL-6), P01375 (TNF), P02768 (ALB), P05412 (JUN), P42574 (CASP3), P04406 (GAPDH), MAPK3 (P27361), P35354 (PTGS2), and MMP9 (P14780).

Two hundred ninety-one intersection target genes were imported into the String database to obtain the PPI dataset, the top 10 Hub target genes of PPI were obtained by Cytoscape. They were P15692 (VEGFA), P05231 (IL-6), P01375 (TNF), P02768 (ALB), P05412 (JUN), P42574 (CASP3), P04406 (GAPDH), P27361 (MAPK3), P35354 (PTGS2), and P14780 (MMP9), respectively, as shown in (Figure 6).

### Outcome Analysis of KEGG Pathways and Biological Processes

**FIGURE 7:**
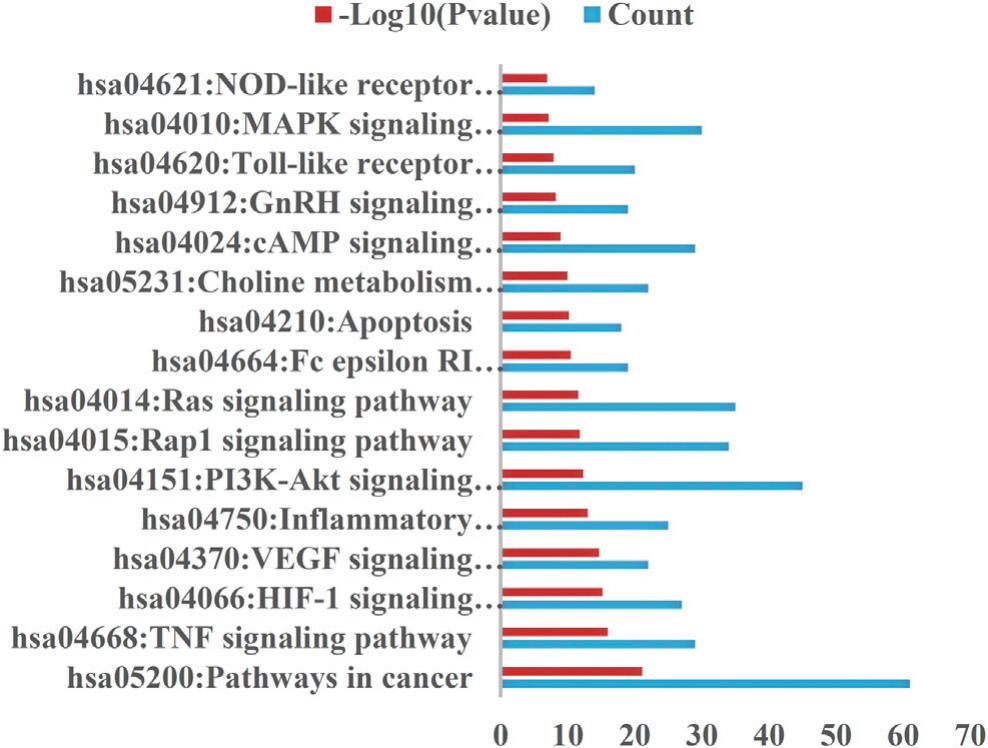
KEGG pathway. Setting the screening conditions of *p* < 0.01 and FDR <0.05, 117 KEGG pathways were obtained from 291 target genes with intersection in DAVID database, and only 16 major pathways are shown in this figure.

**FIGURE 8:**
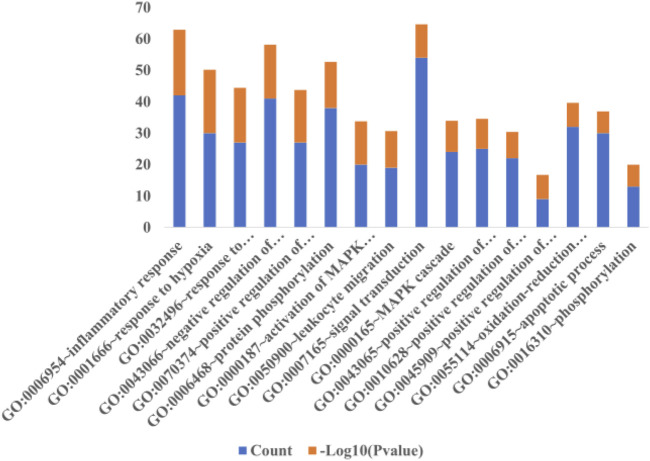
Biological process. When the screening conditions of *p* < 0.01 and FDR <0.05 were set, 337 BP enriched datasets were obtained from 291 intersection target genes in DAVID database, and only 16 major BP results are shown in this figure.

The selection conditions of *p* < 0.01 and FDR <0.05 were set, and 117 KEGG pathways and 337 BP-enriched datasets of 291 target genes with intersection were obtained from DAVID database and were presented by bar plots. KEGG pathways mainly include TNF signaling pathway, HIF-1 signaling pathway, VEGF signaling pathway, PI3K-Akt signaling pathway, and so on (Figure 7). BP mainly includes inflammatory response, response to hypoxia, platelet activation, protein phosphorylation, and so on, as shown in (Figure 8).

### Analysis of Molecular Docking Results

**FIGURE9:**
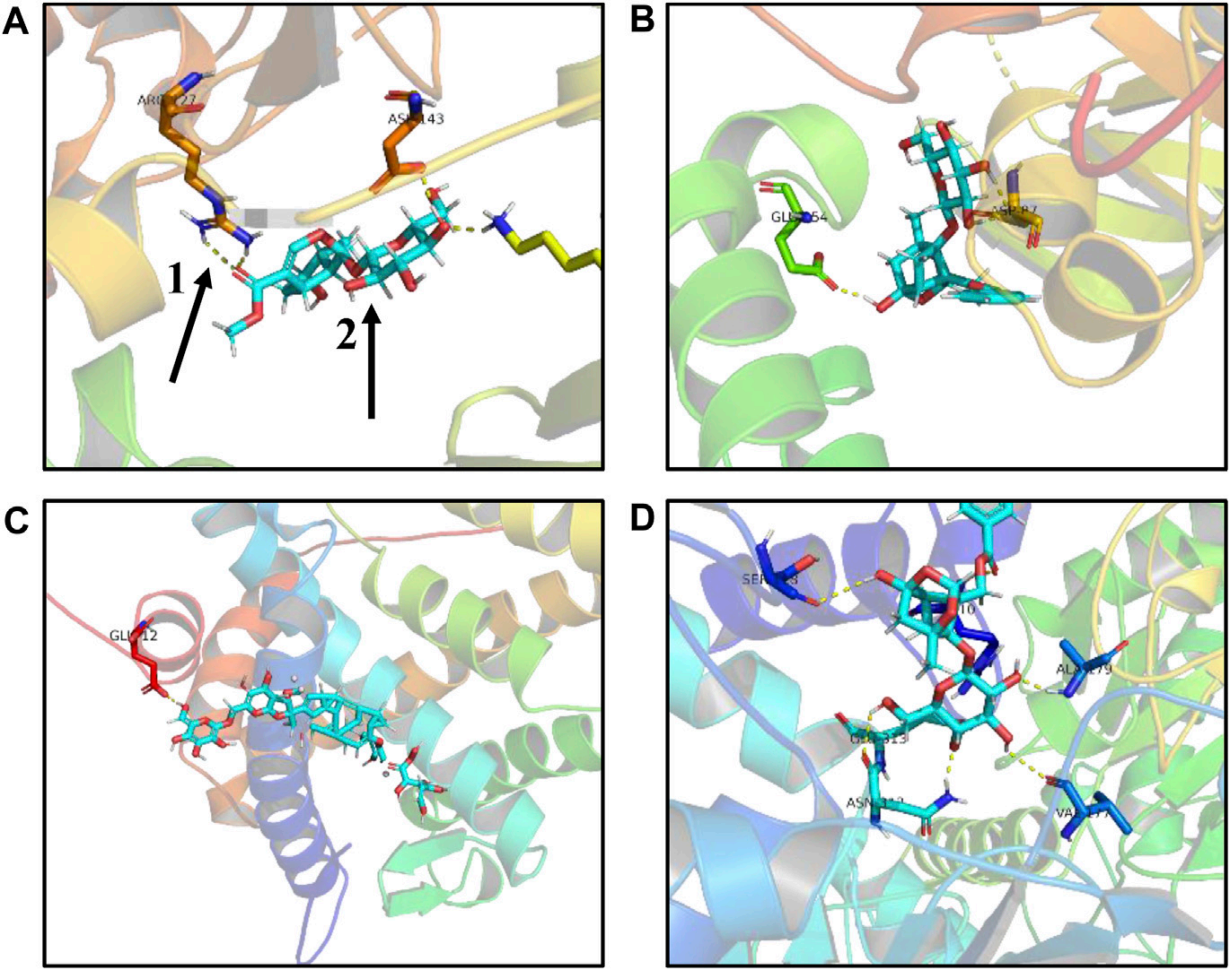
Schematic diagram of molecular docking results. **(A)** Loganin is linked to the SRC active site through two hydrogen bonds to form a complex. **(B)** Paeoniflorin is linked to the SRC active site through two hydrogen bonds to form a complex. **(C)** Ginsenoside Rb1 is linked to the NR3C2 active site through one hydrogen bond to form a complex. **(D)** Paeoniflorin is linked to the GAPDH active site through three hydrogen bonds to form a complex, one for hydrogen bond, and two for small compound ligand.

In the first step, Hub target genes obtained through the MCC and PPI were applied to the PDB database to obtain their protein crystal structures and then uploaded to the DockThor online molecular docking tool. The second step is to upload the 3D structure of small molecule compounds downloaded from PubChem database to the DockThor online tool in the form of sdf. The procedure was as follows: After adding H, click “send to DockThor.” Step 3: Click the blind docking to identify the active site. In the fourth step, click “dock” for molecular docking. Finally, eight pairs of Hub target genes obtained above were successfully docked with their corresponding small molecule compounds, respectively; loganin and paeoniflorin were docked with SRC, ginsenoside Rb1 was docked with NR3C2, paeoniflorin was docked with GAPDH, oleanolic acid and ursolic acid docked with IL-6, paeoniflorin docked with MMP9, and paeoniflorin docked with VEGFA, as shown in (Table 2). According to the docking results and literature reports, three target genes, NR3C2, SRC, and GAPDH, were finally selected for experimental verification. Their docking results with their corresponding small molecule compounds are shown in (Figure 9).

**TABLE 2:** Molecular docking information table.

Genes	PDB ID	Compound	Score
VEGFA	5t89	Paeoniflorin	−7.274
SRC	3el8	Loganin	−6.834
SRC	3el8	Paeoniflorin	−7.417
NR3C2	6ggg	Ginsenoside Rb1	−10.776
MMP9	1gkd	Paeoniflorin	−7.321
IL-6	1alu	Oleanolic acid	−7.543
IL-6	1alu	Ursolic acid	−7.715
GAPDH	1ihy	Paeoniflorin	−8.314

### Analysis of Immunohistochemical Test Results

**FIGURE 10:**
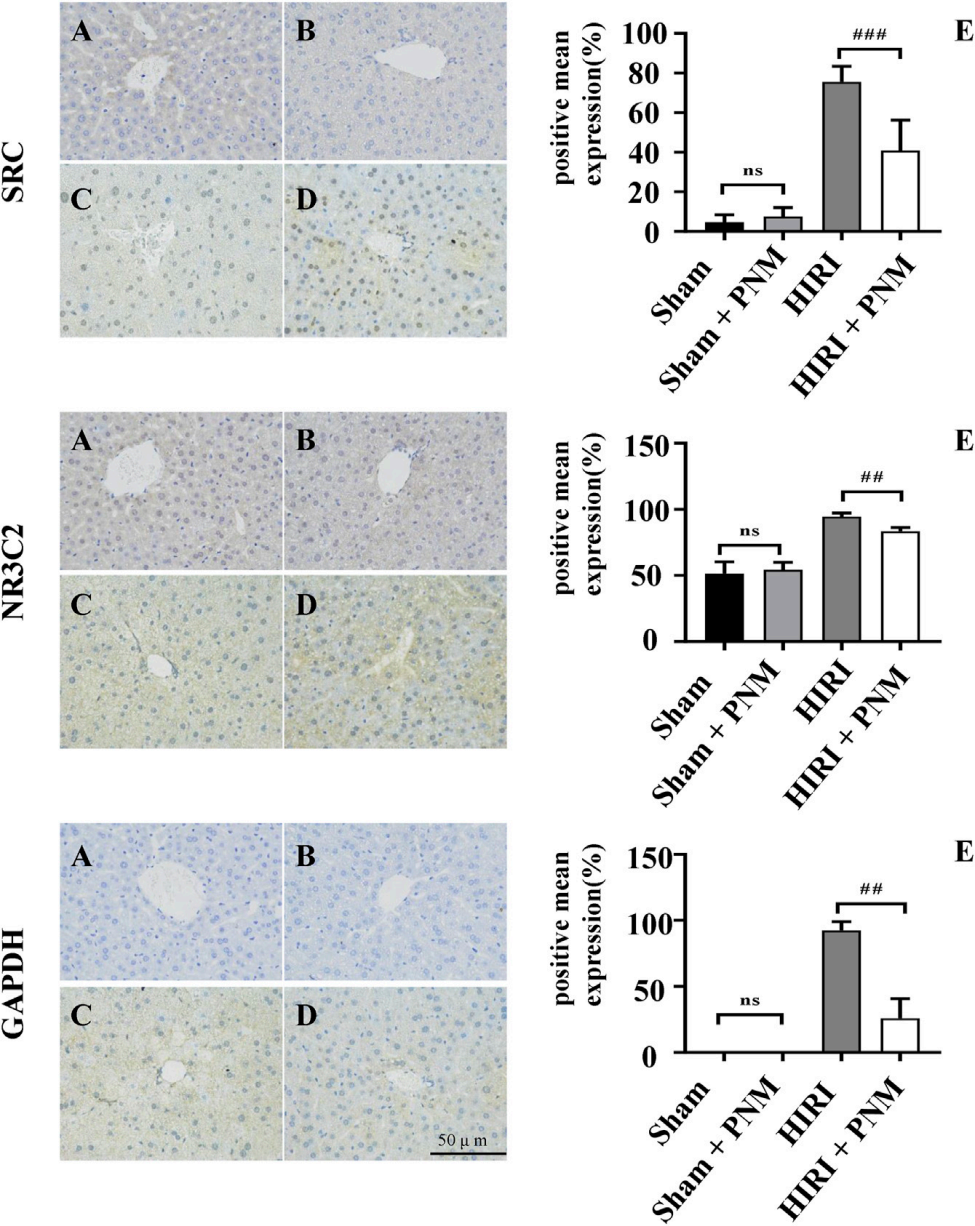
Expression of NR3C2, SRC, and GAPDH in HIRI of mice verified by immunohistochemical assay **(A)** = sham, **(B)** = sham + PNM, **(C)** = HIRI, **(D)** = HIRI + PNM. Diagram scale is 50 μm, magnified 400 times. **(E)** is a bar chart made by analyzing the differences between groups by one-way analysis of variance and Tukey *post hoc* statistical methods according to H-score (n = 3). Bar chart **(E)** shows that the expressions of NR3C2, SRC, and GAPDH were significantly increased in the HIRI group compared with the sham group (##*p* ˂ 0.01). Compared with the HIRI group, the expressions of NR3C2, SRC, and GAPDH in the HIRI + PNM group were significantly decreased (##*p* ˂ 0.01). There was no statistical significance in the expression of the three target genes in the sham and sham + PNM groups (*p* > 0.05). The experimental results showed that PNM protected HIRI in mice by down-regulating the expression of NR3C2, SRC, and GAPDH.

The sections of sham, sham + PNM, HIRI, and HIRI + PNM groups (n = 3) were immunostained with NR3C2 (1:500), SRC (1:500), and GAPDH (1:1,000), respectively. The positive number and staining intensity in each section were converted into corresponding values by H-score, so as to achieve the semiquantitative purpose of tissue staining. H-score is between 0 and 300, and the greater the value, the stronger the comprehensive positive intensity (Guo et al., 2019; Paschalis et al., 2019). The results showed that the expressions of NR3C2, SRC, and GAPDH were significantly increased in the HIRI group compared with the sham group (*p* < 0.001), indicating that the expression of these three target genes was low in normal liver tissue and high in HIRI. Compared with the HIRI group, the expressions of NR3C2, SRC, and GAPDH in the HIRI + PNM group were significantly decreased (*p* < 0.01), indicating that PNM can protect mouse HIRI through the expression of these three target genes. There were no significant differences in the expressions of NR3C2, SRC, and GAPDH in the sham and sham + PNM groups (*p* > 0.05), indicating that PNM did not cause damage to normal liver tissue of mice, as shown in (Figure 10).

## Discussion

Partial hepatectomy and liver transplantation are the most effective methods for the treatment of liver malignant tumors and end-stage liver disease. HIRI is a common pathological process during these operations and one of the important causes of perioperative liver failure and death. Therefore, it is of great significance to explore and intervene in the pathogenesis and defense means of HIRI. Notoginseng, dogwood, and white peony root, which make up PNM, are commonly used Chinese medicines in clinical practice, and they are safe and effective. Modern pharmacological studies have shown that these three drugs have regulatory effects as anti-inflammatory, immunomodulatory, antioxidant, anti-tumor, and so on (Liao et al., 2015; Liu et al., 2021; Chen et al., 2021). Previous animal experiments have shown that the HIRI + PNM group can significantly reduce the expressions of AST and ALT in serum of mice in the HIRI group, and AST and ALT are two of the examination indexes reflecting the physiological function of the liver, and the higher their values are, the more serious the damage of the liver cells is (Asbaghi et al., 2021). As PNM can intervene in the occurrence of HIRI by reducing AST and ALT, we continued to use bioinformation network pharmacology to explore the molecular mechanism of PNM’s protection of HIRI.

Twenty Hub target genes related to HIRI were obtained by applying many online pharmacological public databases and various analysis software, as shown in Figures 5, 6. DockThor online tool was used to carry out molecular docking of these target gene receptors and their corresponding small molecule conjugated ligands, and the results are shown in Table 2. Cao et al. (2017) recently reported that before bone marrow–derived endothelial progenitor cell transplantation, exogenous liposome delivery of VEGF gene may be an effective strategy to reduce orthotopic liver transplantation–induced HIRI. Jiao et al. (2021) reported that VEGF and IL-6 play an important role in the study of the potential of mesenchymal stem cell secretome to promote liver regeneration after hepatic ischemia/reperfusion combined with partial resection. Kato et al. (2014), Huang et al. (2019) reported that inhibition of the expression of MMP9 gene will greatly affect the process of HIRI, indicating that it is a good way to treat or alleviate HIRI research. Therefore, VEGFA, MMP9, and IL-6 have been reported in the studies of liver ischemia/reperfusion injury–related diseases, so the molecular mechanism of these target genes will not be discussed here.

SRC is a nonreceptor tyrosine protein kinase, which is the first proto-oncogene existing in a normal cell state in the body, and plays an important role in maintaining normal physiological functions of the body (Kaplan et al., 1990). The experimental results of this study showed that the expression of immunohistochemical SRC was increased in the HIRI group compared with the sham group (*p* ˂ 0.01). Compared with the HIRI group, the expression of SRC in the PNM + HIRI group was decreased (*p* < 0.01). There was no statistical significance in the expression of SRC in sham and sham + PNM groups (*p* > 0.05). The above experimental results indicate that PNM can reduce the occurrence of HIRI in mice by down-regulating the expression of SRC and the low expression of SRC in normal tissues. This result is consistent with literature reports that SRC is precisely regulated in normal cells and tissues, but is highly expressed in a variety of human tumors, such as lung cancer, colon cancer, nausea and hematologic diseases, and breast cancer (Wang et al., 2021). SRC is a 60-kDa nonreceptor tyrosine kinase family. At present, a total of nine different family members have been found in vertebrate cells. In the SRC kinase family, SRC proto-oncogene is mainly studied, and its changes in the occurrence and development of various diseases are mainly discussed (Wheeler et al., 2009). In this study, the small molecule ligand loganin and paeoniflorin in PNM were connected to the active site of SRC through hydrogen bond to form a complex, thereby inhibiting the activation of SRC to reduce the effect of ischemia/reperfusion injury.

NR3C2 (mineralocorticoid receptor [MR]) is nuclear receptor subfamily 3 group C member 2. It belongs to the adrenocortical receptor family with glucocorticoid receptor. MR is expressed not only in the epithelial tissues of liver and kidney, but also in human and mouse tissues such as large intestine, salivary glands, airway, sweat glands, and inner ear (Viengchareun et al., 2007). Inactivated MR mainly exists in the cytoplasm. Once MR binds to its ligand aldosterone, the MR configuration changes can occur. It can activate the nuclear localization signal, make the activated receptor–ligand complex quickly transfer to the nucleus, participate in the reaction of gene promoter or interact with other transcription factors, and induce the activation or inhibition of transcription and to regulate MR-related signaling pathways and a variety of physiological and pathological responses (Bouarab et al., 2021). Our experimental results showed that compared with the sham group, the expression of MR was increased in the HIRI group (*p* ˂ 0.01). Compared with the HIRI group, the expression of MR in the HIRI + PNM group was significantly decreased (*p* ˂ 0.01). The expression of MR in the sham and sham + PNM groups was not statistically significant (*p* > 0.05), and its expression content was low. The above experimental results indicate that PNM can reduce the occurrence of HIRI in mice by reducing the expression of MR, and the expression of MR is low in normal tissues. Moreover, PNM did not cause damage to normal mouse liver tissue. The expression of MR mRNA in the hypothalamus of Lewis spontaneous kidney-Yang deficiency rats was slightly increased compared with that of normal Wistar rats. Interfering with the Chinese medicine *Cordyceps sinensis* could inhibit the expression of MR mRNA in the hypothalamus of Lewis spontaneous kidney-Yang deficiency rats (Zhang L. et al., 2020). It was found that MR was highly expressed in renal tissues of rats with obstructive nephropathy, and traditional Chinese medicine for invigorating qi and activating blood could reduce the expression of MR in renal tissues by regulating protein kinase-1 (SGK-1) (Chai et al., 2020). These reports are consistent with our findings that inhibiting the expression of MR in tissues can improve the pathophysiological status of the body. In this study, ginsenoside Rb1, a small molecular compound in PNM, was linked to the active site of MR through hydrogen bonds to form a complex, thereby inhibiting the activation of MR to reduce the effect of ischemia/reperfusion injury.

GAPDH is a key enzyme in the glycolytic pathway that catalyzes the conversion of glyceraldehyde 3-phosphate to 1, 3-phosphate glycerate. As a multifunctional enzyme, GAPDH also plays roles in gene expression regulation, DNA repair and replication, neurodegeneration, pathogenic mechanism, regulation of apoptosis and autophagy, and so on (Butera et al., 2019). GAPDH plays an important role in various diseases mainly through aggregation, nuclear translocation, and binding to specific proteins. Our results showed that the expression of GAPDH was increased in the HIRI group compared with the sham group (*p* < 0.01). Compared with the HIRI group, the expression of GAPDH was significantly decreased in the HIRI + PNM group (*p* < 0.01). The expression of GAPDH in the sham and sham + PNM groups was not statistically significant (*p* > 0.05), and its expression content was low. The experimental results indicated that PNM could reduce the occurrence of HIRI in mice by reducing the expression of GAPDH, and the expression of GAPDH was low in normal tissues. The results of this study are consistent with the findings of these scholars on the expression of GAPDH in these diseases. Darusman et al. report that levels of GAPDH were significantly higher in patients with Alzheimer disease neurodegenerative disease than in healthy cynomolgus monkeys (Darusman et al., 2021). Galbiati et al. reported that inhibition of GAPDH expression could be used as a strategy for the development of drugs against various tumors and parasites (Galbiati et al., 2020). Wang et al. reported that GAPDH was highly expressed in lung adenocarcinoma tissues (Wang et al., 2020). Hao et al. reported that their clinical tissue studies showed that GAPDH protein levels were significantly upregulated in lung squamous cell carcinoma tissues compared with the adjacent normal lung tissues, and this was confirmed by Western blotting and immunohistochemistry. *In vitro*, GAPDH knockdown by siRNA can significantly reduce the proliferation, migration, and invasion of lung squamous cell carcinoma cells (Hao et al., 2020). In this study, paeoniflorin, a small molecular compound in PNM formed a complex with the active site of GAPDH through hydrogen bond, which effectively inhibited the expression of GAPDH in mouse HIRI and alleviated the occurrence of HIRI. However, whether the decrease of GAPDH expression is caused by inhibiting its aggregation, nuclear translocation, or preventing the binding with specific proteins still needs further discussion.

## Conclusion

In short, HIRI is an urgent clinical problem to be solved, so it is of great clinical significance to carry out relevant research. In this study, small molecular compounds in PNM, such as ginsenoside Rb1, paeoniflorin, and loganin, formed complexes with NR3C2, GAPDH, and SRC, respectively, to reduce the expression of these target genes in HIRI, thus achieving the protection of mouse HIRI. There are many targets associated with the occurrence of HIRI. Our study confirmed that NR3C2, GAPDH, and SRC are also important target genes in the prevention and treatment of HIRI. In the future, we will further explore the molecular mechanism of these target genes against HIRI. Inhibitors through NR3C2, GAPDH, and SRC will be developed by ginsenoside Rb1, paeoniflorin, and loganin, making these three target genes promising target genes to weaken or stop the development of HIRI disease.

## Data Availability

The original contributions presented in the study are included in the article/Supplementary Material, further inquiries can be directed to the corresponding author.
