# Supplementary material for: The Protective Effect of Panax notoginseng Mixture on Hepatic Ischemia/Reperfusion Injury in Mice via Regulating NR3C2, SRC, and GAPDH
Source: Front Pharmacol. 2021 Nov 11;12:756259. doi: 10.3389/fphar.2021.756259 (PMC8632037; doi:10.3389/fphar.2021.756259)
Supplement: Supplementary file 1 [file Table1.DOCX]

**TABLE 1 Basic information of PNM compounds**

| **Compound code** | **Compound name** | **OB/%** | **DL** | **medicine** |
| --- | --- | --- | --- | --- |
| MOL008457 | Tetrahydroalstonine | 32.42 | 0.81 | Dogwood |
| MOL007487 | Notoginsenosider1 | 5.42 | 0.13 | Notoginseng |
| MOL007488 | Notoginsenosider2 | 7.69 | 0.28 | Notoginseng |
| MOL007476 | Ginsenoside Rb1 | 6.29 | 0.04 | Notoginseng |
| MOL007475 | Ginsenoside F2 | 36.43 | 0.25 | Notoginseng |
| MOL005531 | Telocinobufagin | 69.99 | 0.79 | Dogwood |
| MOL005530 | Hydroxygenkwanin | 36.47 | 0.27 | Dogwood |
| MOL005503 | Cornudentanone | 39.6 | 0.33 | Dogwood |
| MOL005489 | 3,6-Digalloylglucose | 31.42 | 0.66 | Dogwood |
| MOL005486 | 3,4-Dehydrolycopen-16-al | 46.64 | 0.49 | Dogwood |
| MOL005481 | 2,6,10,14,18-Pentaene | 33.40 | 0.24 | Dogwood |
| MOL005360 | Malkangunin | 57.71 | 0.63 | Dogwood |
| MOL005344 | Ginsenoside rh2 | 36.32 | 0.56 | Notoginseng |
| MOL003137 | Leucanthoside | 32.12 | 0.78 | Dogwood |
| MOL002883 | Ethyl oleate (NF) | 32.40 | 0.19 | Dogwood |
| MOL002879 | Diop | 43.59 | 0.39 | Dogwood, Notoginseng |
| MOL001933 | Oxypaeoniflorin | 21.88 | 0.78 | White paeony root |
| MOL001930 | Benzoyl paeoniflorin | 31.77 | 0.75 | White paeony root |
| MOL001928 | Albiflorin_qt | 66.64 | 0.33 | White paeony root |
| MOL001927 | Albiflorin | 12.09 | 0.77 | White paeony root |
| MOL001925 | Paeoniflorin_qt | 68.18 | 0.40 | White paeony root |
| MOL001924 | Paeoniflorin | 53.87 | 0.79 | White paeony root |
| MOL001921 | Lactiflorin | 49.12 | 0.80 | White paeony root |
| MOL001911 | Albiflorin R1 | 21.29 | 0.82 | White paeony root |
| MOL000874 | Paeonol | 28.74 | 0.04 | White paeony root |
| MOL001792 | DFV | 32.76 | 0.18 | Notoginseng |
| MOL001771 | Poriferast-5-en-3beta-ol | 36.91 | 0.75 | Dogwood |
| MOL001680 | Loganin | 59.00 | 0.44 | Dogwood |
| MOL001495 | Ethyl linolenate | 46.10 | 0.20 | Dogwood |
| MOL001494 | Mandenol | 42.00 | 0.19 | Dogwood, Notoginseng |
| MOL000554 | Gallicacid-3-O-(6'-O-galloyl)-glucoside | 30.25 | 0.67 | Dogwood |
| MOL000511 | Ursolic acid | 16.77 | 0.75 | Dogwood |
| MOL000492 | (+)-Catechin | 54.8 | 0.24 | White paeony root |
| MOL000449 | Stigmasterol | 43.8 | 0.75 | Dogwood, Notoginseng |
| MOL000422 | Kaempferol | 41.88 | 0.24 | White paeony root |
| MOL000359 | Sitosterol | 36.91 | 0.75 | White paeony root |
| MOL000358 | Beta-sitosterol | 36.90 | 0.75 | White paeony root, Dogwood, Notoginseng |
| MOL000263 | Oleanolic acid | 29.02 | 0.76 | Whitepaeonyroot, Dogwood |
| MOL000211 | Mairin | 55.38 | 0.24 | White paeony root |
| MOL000098 | Quercetin | 46.43 | 0.28 | Notoginseng |
| MOL000069 | Palmitic acid | 19.30 | 0.10 | Dogwood |
