# Supplementary material for: The Protective Effect of Panax notoginseng Mixture on Hepatic Ischemia/Reperfusion Injury in Mice via Regulating NR3C2, SRC, and GAPDH
Source: Front Pharmacol. 2021 Nov 11;12:756259. doi: 10.3389/fphar.2021.756259 (PMC8632037; doi:10.3389/fphar.2021.756259)
Supplement: Supplementary file 2 [file Table2.DOCX]

| **Genes** | **PDB ID** | **Compound** | **Score** |
| --- | --- | --- | --- |
| VEGFA | 5t89 | Paeoniflorin | -7.274 |
| SRC | 3el8 | Loganin | -6.834 |
| SRC | 3el8 | Paeoniflorin | -7.417 |
| NR3C2 | 6ggg | Ginsenoside Rb1 | -10.776 |
| MMP9 | 1gkd | Paeoniflorin | -7.321 |
| IL6 | 1alu | Oleanolic acid | -7.543 |
| IL6 | 1alu | Ursolic acid | -7.715 |
| GAPDH | 1ihy | Paeoniflorin | -8.314 |

**TABLE.2 Molecular docking information table**
