# Supplementary material for: The Protective Effect of Panax notoginseng Mixture on Hepatic Ischemia/Reperfusion Injury in Mice via Regulating NR3C2, SRC, and GAPDH
Source: Front Pharmacol. 2021 Nov 11;12:756259. doi: 10.3389/fphar.2021.756259 (PMC8632037; doi:10.3389/fphar.2021.756259)
Supplement: Supplementary file 3 [file Table3.DOCX]

| **Full Name** | **abbreviation** |
| --- | --- |
| Panax notoginseng mixture | PNM |
| Hepatic Ischemia /Reperfusion Injury | HIRI |
| Aspartate transaminase | AST |
| Alanine aminotransferase | ALT |
| Glyceraldehyde-3-phosphate dehydrogenase liver | GAPDH |
| Tyrosine-protein kinase | SRC |
| Mineralocorticoid receptor | NR3C2 |
| Interleukin-6 | IL6 |
| Vascular endothelial growth factor A | VEGFA |
| Matrix metalloproteinase 9 | MMP9 |
| Traditional Chinese Medicine | TCM |
| Oral bioavailability | OB |
| Drug-like drug | DL |
| Kyoto Encyclopedia of Genes and Genomes | KEGG |
| Biological pathways | BP |
| Hematoxylin and Eosin | HE |
| Histochemistry Score | H-Score |
| Protein-protein interaction | PPI |
